# Regional homogeneity alterations reflect pain and emotional dysregulation in adenomyosis

**DOI:** 10.1080/07853890.2026.2620857

**Published:** 2026-01-30

**Authors:** Wenjiang Wei, Yanying Chen, Kelei Hua, Bin Xia, Rujin Li, Weizhao Lin, Zichao Chen, Wenqing Xiao, Kanghui Yu, Yi Yin, Shoujun Xu, Yunfan Wu

**Affiliations:** a Department of Interventional Vascular, The Affiliated Guangdong Second Provincial General Hospital of Jinan University, Guangzhou, PR China; b Department of Medical Imaging, The Affiliated Guangdong Second Provincial General Hospital of Jinan University, Guangzhou, PR China; c Guangdong Medical University, Zhanjiang, PR China; d The Second School of Clinical Medicine, Southern Medical University, Guangdong Second Provincial General Hospital, Guangzhou, PR China; e Department of Radiology, Jieyang People’s Hospital, Jieyang, PR China; f Department of Radiology, Shenzhen Children’s Hospital, Shenzhen, PR China

**Keywords:** Static, dynamic, regional homogeneity, adenomyosis, dysmenorrhea, pain, anxiety, depression, empathy

## Abstract

**Purpose:**

Adenomyosis (AM) with dysmenorrhea (AMD) is a global public health concern that may involve abnormal brain function and heightened vulnerability to anxiety and depression. This study aimed to investigate static and dynamic regional homogeneity (sReHo and dReHo) alterations in AMD and their associations with clinical symptoms.

**Methods:**

Fifty-two patients with AMD and 52 age- and education-matched healthy controls (HCs) underwent resting-state functional magnetic resonance imaging (rs-MRI). sReHo and dReHo maps were generated and compared using two-sample *t*-tests (voxel-level *p* < 0.01; cluster-level, Gaussian random field corrected *p* < 0.05), with age, education and head motion as covariates. Effect sizes (Cohen’s *d*) were calculated for significant clusters. Partial correlation analyses assessed relationships between regional homogeneity (ReHo) alterations and clinical measures, controlling for medication use, menstrual phase and psychiatric history.

**Results:**

Compared with HCs, AMD patients showed significantly increased sReHo and dReHo in the right fusiform gyrus (FFG; *d* = 0.84, *p* < 0.001) and decreased values in the bilateral supramarginal gyri (SMG; *d* = −0.73, *p* < 0.001). Additional regiongs exhibiting abnormal dReHo were included the bilateral angular gyri, right hippocampus, right cerebellum (lobule 4_5) , left middle frontal gyrus (MFG) and multiple subdivisions of the inferior frontal gyrus. Importantly, ReHo in the right FFG positively correlated with pain severity (visual analogue scale (VAS): *r* = 0.539-0.797, *p* < 0.001) and anxiety (Hamilton Anxiety Scale (HAMA): *r* = 0.442, *p* = 0.001). Abnormalities in the right hippocampus and cerebellum were also significantly associated with anxiety and depression scores (*r* = 0.555–0.861, all *p* < 0.001).

**Conclusions:**

AMD is characterized by altered intrinsic brain activity in pain- and emotion-related regions, supporting a central mechanism underlying chronic pain and affective symptoms. These findings provide neuroimaging evidence for AMD-related brain dysfunction. Longitudinal studies with larger cohorts and hormonal control are warranted to confirm these results and clarify causal relationships.

## Introduction

Adenomyosis (AM) is characterized by invasive endometrial glands and stroma in the myometrium with surrounding myometrial hyperplasia [1]. Prevalence varies markedly by age, ethnicity and uterine surgery history, and the prevalence of AM was 8.8–88% [2]. This wide variability is largely due to differences in diagnostic approaches (e.g. histopathological vs. imaging-based diagnosis), heterogeneity in study populations, and geographic or ethnic factors influencing incidence rates. Adenomyosis can cause severe clinical manifestations, such as progressive dysmenorrhea, abnormal uterine bleeding, subfertility, miscarriage and other obstetric complications. Dysmenorrhea and abnormal uterine bleeding are the most common reasons prompting patients to seek medical attention [3]. Adenomyosis can be managed through a variety of medical and surgical approaches. Medical therapy is often preferred for patients who wish to preserve their uterus or future fertility, those approaching menopause, or individuals with contraindications to surgery. Currently, hormonal agents – such as gonadotropin‑releasing hormone analogues, progestins and combined oral contraceptives – constitute first‑line treatment. These therapies can alleviate pain symptoms and achieve modest reductions in uterine volume, but they are associated with dose‑dependent adverse effects, and symptom recurrence is virtually inevitable once treatment is discontinued. Notably, AM is highly prevalent among patients undergoing hysterectomy, affecting up to 42.0% of such cases [4]. In light of the limited clinical treatment options available for AM coupled with severe dysmenorrhea, patients often opt for hysterectomy to alleviate their menstrual pain. While this choice may have implications for fertility, it vividly underscores the profound impact of AM on women’s quality of life. Furthermore, the profound dysmenorrhea linked to AM can lead to extensive repercussions, such as disturbances in daily activities, appetite and sleep patterns, along with an elevated susceptibility to anxiety and depression [5,6]. Despite being a benign gynaecological condition, AM complicated by severe dysmenorrhea imposes a considerable healthcare burden and diminishes overall well-being [7,8]. It is estimated that women with symptomatic AM may lose up to 9–12 working days annually due to pain and associated symptoms, contributing to substantial direct and indirect healthcare costs. On the other hand, previous studies [9–11] have proposed that the cyclical release of inflammation and neurogenic pain mediators plays a role in spinal pain hypersensitivity and central sensitization in AM-related dysmenorrhea. Nevertheless, the precise pathogenic mechanism responsible for dysmenorrhea in AM remains incompletely understood [8,12]. Due to the elusive pathogenesis and complex clinical presentations of AM with dysmenorrhea (AMD), which impose substantial burdens on both patients and their families, it remains an important global public health issue [13].

Resting-state functional magnetic resonance imaging (rs-fMRI) provides a non-invasive approach to studying spontaneous brain activity. Among its quantitative metrics, regional homogeneity (ReHo) assesses the synchronization of neural activity in a voxel and its immediate neighbours, measured by Kendall’s coefficient of concordance [14]. ReHo can be analysed from both static and dynamic perspectives. In a study conducted by Jiao et al. abnormal static ReHo (sReHo) was observed primarily in the bilateral cerebellum, left inferior frontal gyrus, medial prefrontal cortex and posterior cingulate gyrus [15]. These regions – including the cerebellum, inferior frontal gyrus, medial prefrontal cortex and posterior cingulate cortex – serve as critical hubs for nociceptive processing, motor coordination, emotional regulation and default‑mode network activity, respectively. Dysregulated synchrony within these circuits may therefore underlie the chronic pelvic pain and affective disturbances observed in AMD. This study underscores the capability of sReHo in detecting aberrant brain function in patients with AM, potentially providing objective imaging evidence to elucidate the neural mechanisms underlying pain symptoms in AM. While sReHo can identify abnormal local activity patterns in static brain function, dynamic ReHo (dReHo) offers further insights into the neuropathological mechanisms of diseases due to its enhanced sensitivity to temporal variations in internal brain tissue dynamics [16]. This dynamic analysis is particularly valuable in chronic pain research, as it reflects the brain’s adaptability and altered temporal coordination under pathological conditions. Currently, dReHo analysis has been extensively applied in exploring the neuropathogenesis of various diseases, including Alzheimer’s disease [17], Parkinson’s disease [18] and post-traumatic stress disorder [19]. However, there is a paucity of research investigating alterations in dReHo in patients with AMD.

The objective of this study is to explore alterations in both static and dynamic regional homogeneity (sReHo and dReHo) in patients with AMD and to examine their relationships with clinical symptoms. Given the current lack of dReHo studies in AMD, our hypotheses are informed by previous findings from sReHo studies in AMD and dReHo alterations in other chronic pain conditions. Specifically, we hypothesize that: (1) AMD patients will exhibit abnormal sReHo and dReHo in core pain- and emotion-related brain regions – including the insula, anterior cingulate cortex (ACC), prefrontal cortices, hippocampus and thalamus; and (2) these neural alterations will be significantly associated with the severity of dysmenorrhea and levels of anxiety and depression. Through this investigation, we aim to deepen understanding of the central mechanisms underlying pain and affective disturbances in AMD, and to explore the potential of ReHo metrics as neuroimaging biomarkers for individualized assessment and therapeutic guidance.

## Materials and methods

### Subjects

This study adhered to the Declaration of Helsinki. All patients were hospitalized for uterine artery embolization surgery in the interventional vascular department of the affiliated Guangdong Second Provincial General Hospital of Jinan University.

This study was approved by the affiliated Guangdong Second Provincial General Hospital Human Research Ethics Committee (Ethical Number: 2024-KY-KZ-087-02), and all participants provided written informed consent. From April 2024 to March 2025, we recruited 55 AMD patients and 54 age-, sex- and education level-matched healthy control (HC) participants. All study subjects were 18–50 years of age and right handed, and all AMD participants were inpatients undergoing uterine artery embolization surgery in the Interventional Vascular Department of the Guangdong Second Provincial General Hospital diagnosed with AM by gynaecologists and interventional physicians. The inclusion criteria for patients with AMD were as follows: (1) diagnosed with AM according to pelvic magnetic resonance imaging (MRI) and (or) gynaecological ultrasound; (2) without fertility requirements but accompanied by progressively worsening dysmenorrhea and other symptoms such as excessive and (or) prolonged menstruation; (3) dysmenorrhea lasting for at least 6 months with average visual analogue scale (VAS) score for pain equal to or greater than 4 (0 = no pain and 10 = worst pain imaginable) during the three months before enrolment; (4) inadequate or intolerable response to analgesics, hormonal treatments or the Mirena IUD, with voluntary acceptance of the need for uterine embolization and an understanding of potential complications. (5) Requiring uterine embolization treatment and understanding the possible complications. All participants in the HC group were recruited from the community and exhibited no progressive dysmenorrhea, abnormal uterine bleeding, or other gynaecological or obstetric symptoms.

The exclusion criteria for both groups were as follows: (1) pregnancy or lactation; (2) pelvic organ diseases other than AM; (3) pituitary gland diseases; (4) history of neurological or psychiatric disorders; (5) other severe life-threatening diseases; (6) brain trauma or history of brain surgery; (7) history of alcohol or drug abuse; (8) oral contraceptive or hormonal supplement use within 6 months prior to the study; (9) participants with abnormal lifestyle patterns, including excessive (>10 h/week) or insufficient (<2 h/week) physical activity, significant sleep disturbances (DSM-5 diagnosis or PSQI >7), or notable dietary changes (prescribed diet or >30% caloric change within 3 months). (10) Significant brain tissue abnormalities identified on routine MRI T1-weighted imaging (T1WI) or FLAIR sequences; (11) any contraindications to MRI.

Clinical data for the AMD group were obtained from electronic medical records between April 2024 and March 2025 or assessed directly at enrolment. Data for the HC group were assessed directly at enrolment from April 2024 to March 2025. All participants underwent MRI scanning during the follicular phase of the menstrual cycle, and surgical procedures were performed within 8 h after the completion of the functional MRI examination. Recent studies [3,20] have suggested that serum CA125, as a biomarker, may be elevated in patients with AM accompanied by dysmenorrhea, and such elevations may be associated with the severity of pain, inflammatory responses and disease progression. All patients with AMD also received pelvic and comprehensive laboratory examinations, including blood haemoglobin and serum CA125 assays, before 24 h of fMRI examination. Laboratory blood tests were not conducted in the HC group.

All participants underwent assessment of their emotional state using the Hamilton Anxiety Scale (HAMA) and Hamilton Depression Scale (HAMD). Additionally, the severity of pain was evaluated using a VAS prior to undergoing MRI scanning.

### MRI acquisition

Resting-state MRI data were acquired on a Philips Ingenia 3.0-T MR scanner (Amsterdam, Netherlands) with 32-channel phased-array head coil at the Department of Medical Imaging, Guangdong Second Provincial General Hospital. All subjects were scanned in the supine position, and their heads were fixed with belts and foam pads as described previously [21,22]. Earplugs were worn to reduce the effects of noise. Functional MRI datasets were obtained with an gradient-echo planar imaging (EPI) sequence with the following acquisition parameters: repetition time (TR), 2000 ms; echo time (TE), 30 ms; axial slices, 33; flip angle (FA), 90°; slice thickness, 3.5 mm with no gap; matrix, 64 × 64; field of view (FOV), 230 mm × 230 mm. A total of 240 volumes were acquired in approximately 8 min. Individual three-dimensional T1-weighted images (T1WIs) were also acquired using an EPI sequence with the following parameters: TR, 25 ms; TE, 4.1 ms; FA, 30°; slice thickness, 1.0 mm with no gap; FOV, 230 mm × 230 mm; matrix, 230 mm × 230 mm; 160 sagittal slices. All subjects were also examined by T2 FLAIR sequences to exclude obvious brain lesions. Images were examined by two physicians with ≥15 years of experience.

### MRI imaging analysis

Images were preprocessed and analysed using the DPARSFA 5.3 Advanced Edition plugin for DPABI 6.2_220915 (http://rfmri.org/dpabi) [23]. Preprocessing included the following steps: (1) removal of the first 10 scans to eliminate the influence of unstable magnetization. (2) Timing correction and removal of images with head movement greater than 1.5 mm in translation or 1.5° in rotation; (3) registration of the remaining images to the standard Montreal Neurological Institute (MNI) template using the DARTEL method, and resampling at a voxel resolution of 3 × 3 × 3 mm^3^; (4) linear detrending and regression of nuisance covariates (the Friston-24 head motion parameters, white matter signal and cerebrospinal fluid signal); (5) band-pass filtering at 0.01–0.08 Hz. During all preprocessing steps, two radiologists with ≥15 years of experience checked the images to ensure segmentation quality and correct registration.

### Computation of static and dynamic regional homogeneity

Static ReHo values were estimated by calculating the KCC of each voxel using DPABI software as reported in previous studies [14]. A sReHo map was then constructed for each subject and transformed into a standardized *z*-score map.

Dynamic ReHo values were calculated using temporal dynamic analysis toolkits based on DPABI V6.2 [24]. A sliding window of 30 TRs (60 s) and a shift step of 1 TR (2 s) were used to calculate the temporal variability of ReHo as reported in previous studies [17,19,25]. For each sliding window, an ReHo graph was constructed to estimate dReHo. Mean dReHo of the whole brain was calculated to normalize each voxel of the dReHo map by *z*-transformation. Finally, sReHo and dReHo maps were smoothed using an 8 mm full-width at half maximum Gaussian kernel prior to statistical analyses.

### Statistical analysis

Datasets were first assessed for normality using the Shapiro–Wilk test. Based on the results, group differences in age, education, VAS scores, HAMA scores, HAMD scores and other clinical data were compared between AMD and HC groups by independent samples *t*-tests with *p* < 0.05 (two-tailed) considered statistically significant. The differences in ReHo between ADD patients and healthy subjects, as well as the association between abnormal ReHo values and performance levels on the VAS, HAMA and HAMD scores were evaluated using the DPARSFA 5.3 Advanced Edition plugin for DPABI 6.2_220915 (http://rfmri.org/dpabi) [23]. Mean *z*-transformed ReHo maps were compared between groups using two-tailed independent samples *t*-tests, with voxel-level significance set at *p* < 0.01 and cluster-level significance set at *p* < 0.05. Results were corrected using the Gaussian random field method. Mean sReHo and dReHo values for all voxels differing significantly between groups were extracted separately using the resting-state fMRI Data Analysis Toolkit (REST; http://resting-fmri.sourceforge.net) and expressed in MNI coordinates. Finally, associations between clinical variables and mean regional dReHo *z*-values of patients with AMD differing significantly from HCs were evaluated by partial correlation tests with age, sex and years of education as covariates. A *p* < 0.05 was considered statistically significant for all correlation coefficients.

## Results

### Demographic and clinical features

The final imaging and correlation analyses included 52 patients with AMD and 52 HC subjects. One AMD patient and two HC subjects were excluded due to MRI intolerance, and another two patients with AMD were excluded due to anatomical brain abnormalities on MRI. The serum CA125 level in patients with AM is higher than the normal range, while the average haemoglobin level is lower than normal. There were no significant differences in age and education between groups (both *p* > 0.05), while HAMA and HAMD scale scores were significantly higher in patients with AMD than the HC group (*p* < 0.05) (Table 1).

**Table 1.** Baseline characteristics of AMD patients and HC subjects.

Characteristic	AMD patients (*n* = 52)	HC participants (*n* = 52)	*p* Value
Age (years)	40.58 ± 4.88	29.56 ± 5.60	0.667
Menstrual phase (day)	4–13	4–7	0.001
During of dysmenorrhea (years)	6.42 ± 5.60	NA	
VAS (score)	8 (4, 10)	NA	
HAMA (score)	20.48 ± 4.46	3.94 ± 2.44	<0.001
HAMD (score)	22.79 ± 4.43	4.94 ± 3.98	<0.001
Serum Ca125 (U/mL)	87 (58, 181)	NA	
Haemoglobin (g/L)	103.35 ± 23.74	NA	

AMD: adenomyosis-associated-dysmenorrhea; HC: healthy controls; VAS: the average visual analogue score; HAMA: the Hamilton rating scales of anxiety; HAMD: the Hamilton rating scales of depression.

Data are mean ± standard deviation; the menstrual phase is maximum and minimum values. Serum CA125 levels were measured, with a reference range of 0–35 U/mL.

### Group differences in sReHo

**Figure 1.**
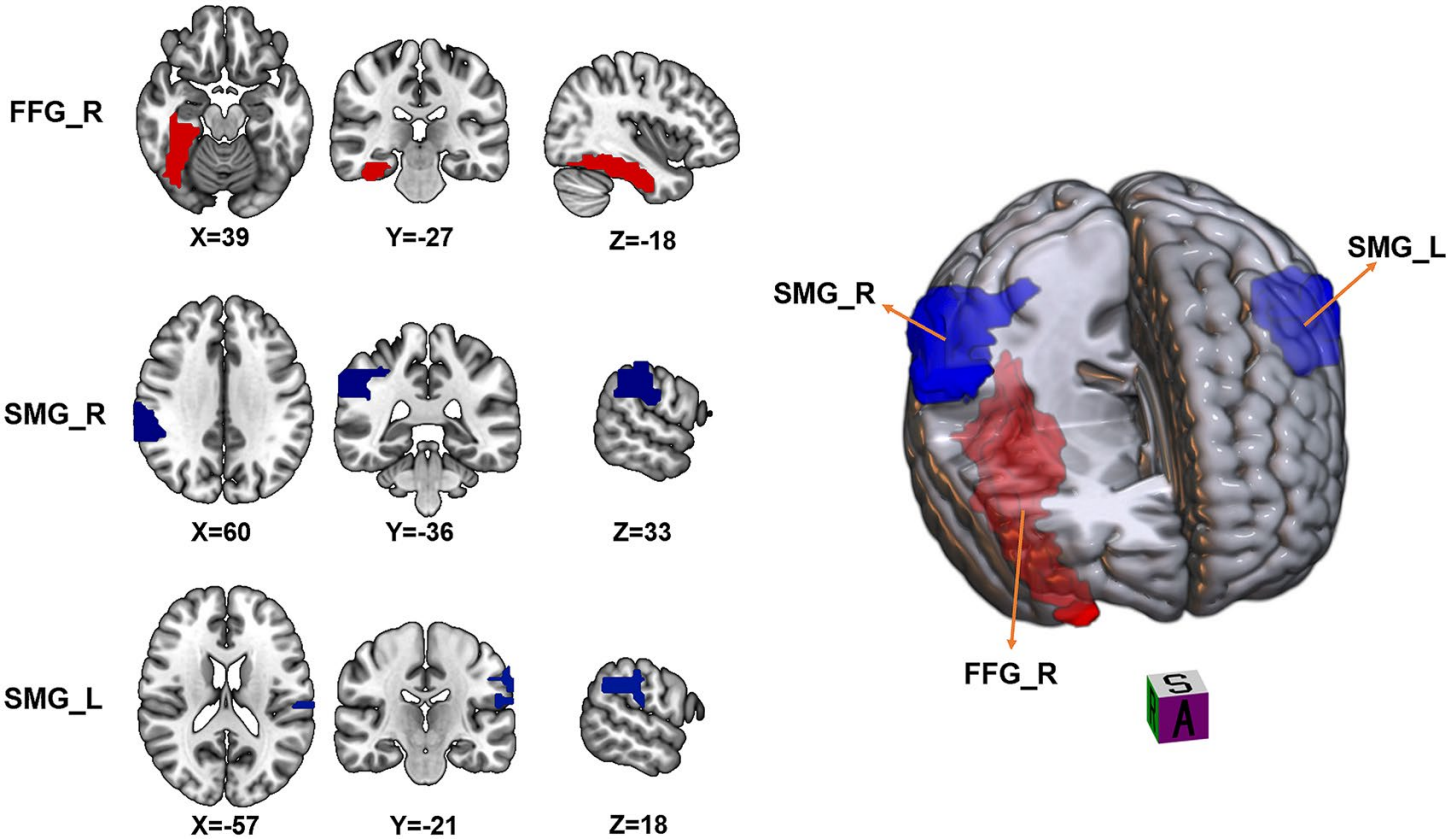
Brain areas with significant sReHo differences between adenomyosis with dysmenorrhea (AMD) patients and healthy control (HC) groups. Regions in red are brain areas where sReHo was significantly greater in AMD group compared to HC group. Regions in blue are brain areas where sReHo was significantly lower in AMD group compared to HC group. The results were multiple compared at the voxel-level (two-tailed voxel-level: *p* < 0.01, Gaussian random field correction, cluster-level: *p* < 0.05).

Compared to the HC group, patients with AMD exhibited significantly greater sReHo values in the right fusiform gyrus (FFG; *p* < 0.001) and lower sReHo values in the bilateral supramarginal gyrus (SMG; *p* < 0.001) (Table 2; Figure 1).

**Table 2.** Altered sReHo and dReHo regions in adenomyosis-associated-dysmenorrhea patients.

Indices	Brain region	MNI coordinate	Voxels	Peak intensity
sReHo	FFG_R	39, −27, −18	188	6.373
	SMG_R	60, −36, 33	155	−5.906
	SMG_L	−57,−21, 18	185	−5.656
dReHo	ANG_R	50, −48, 32	98	−5.592
	ANG_L	−42, −63, 39	100	−6.662
	SMG_R	60, −33, 39	145	−5.596
	SMG_L	−61, −33, 32	114	−6.162
	Cerebelum_4_5_R	24, −42, −30	130	4.556
	FFG_R	39, −33, −21	170	4.939
	hippocampus_R	33, −36, −3	64	4.615
	MFG_L	39, 30, 18	160	5.085
	ORBinf_L	−27, 21, −24	75	−4.181
	IFGtriang_L	−45, 27, 18	94	−4.370
	IFGoperc_L	−39, 12, 30	71	−5.367

FFG: fusiform gyrus; ANG: angular gyrus; SMG: supramarginal gyrus; MFG: middle frontal gyrus; ORBinf: orbital part of inferior frontal gyrus; IFGtriang: triangular part of inferior frontal gyrus; IFGoperc: opercular part of inferior frontal gyrus; L: left; R: right.

### Group differences in dReHo

**Figure 2.**
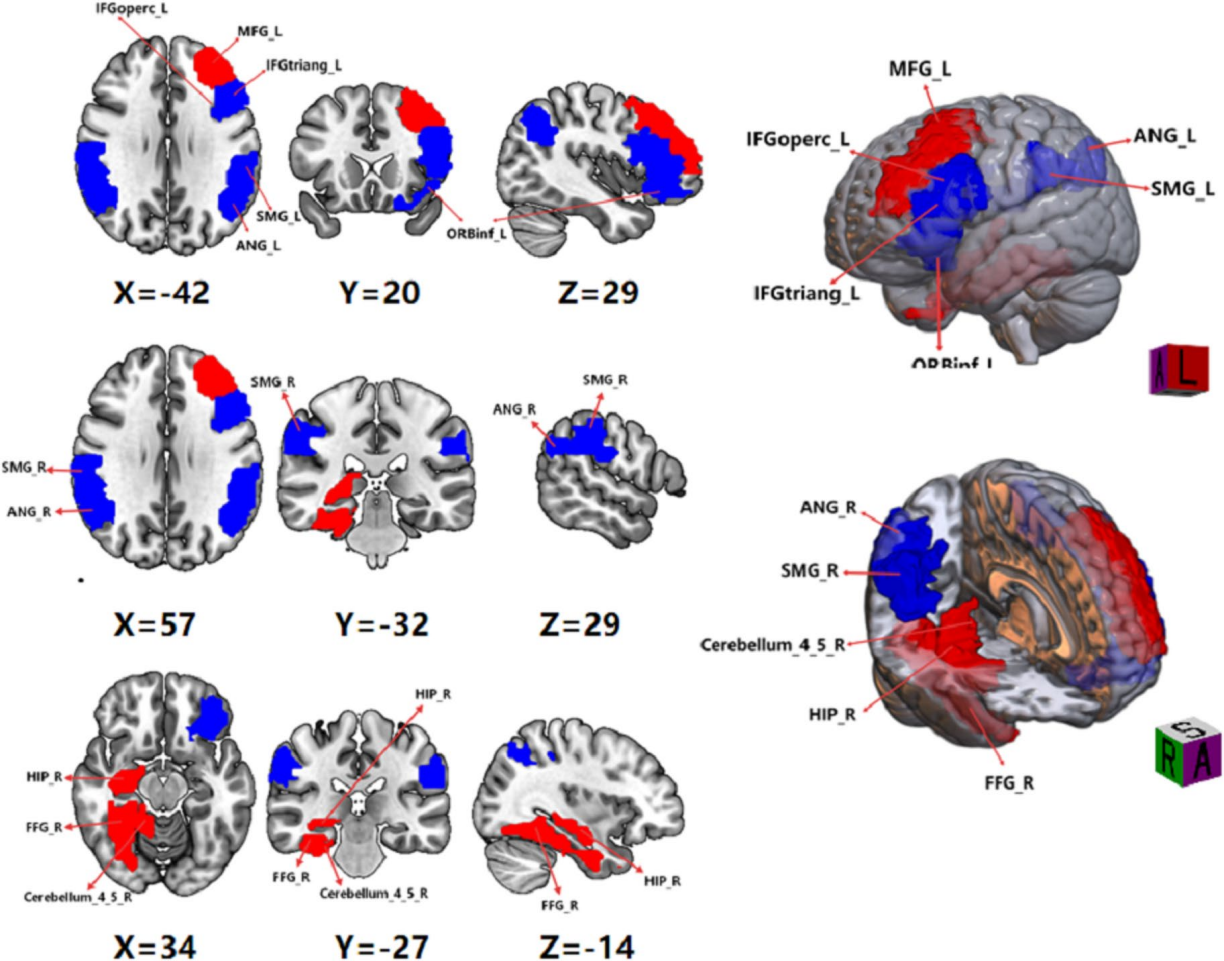
Brain areas with significant dReHo differences between adenomyosis with dysmenorrhea (AMD) patients and healthy control (HC) groups. Regions in red are brain areas where dReHo was significantly greater in AMD group compared to HC group. Regions in blue are brain areas where sReHo was significantly lower in AMD group compared to HC group. The results were multiple compared at the voxel-level (two-tailed voxel-level: *p* < 0.01, Gaussian random field correction, cluster-level: *p* < 0.05).

Compared to the HC group, patients with AMD exhibited significantly greater dReHo in the right FFG, and right cerebellum_4_5, right hippocampus, left middle frontal gyrus (MFG). In contrast, reduced dReHo was found in multiple brain regions, including bilateral SMG, bilateral angular gyrus (ANG), left orbital part of the inferior frontal gyrus (ORBinf), left triangular part of the inferior frontal gyrus (IFGtriang) and left opercular part of the inferior frontal gyrus (IFGoperc) (Table 2; Figure 2).

### Correlation analysis

**Figure 3.**
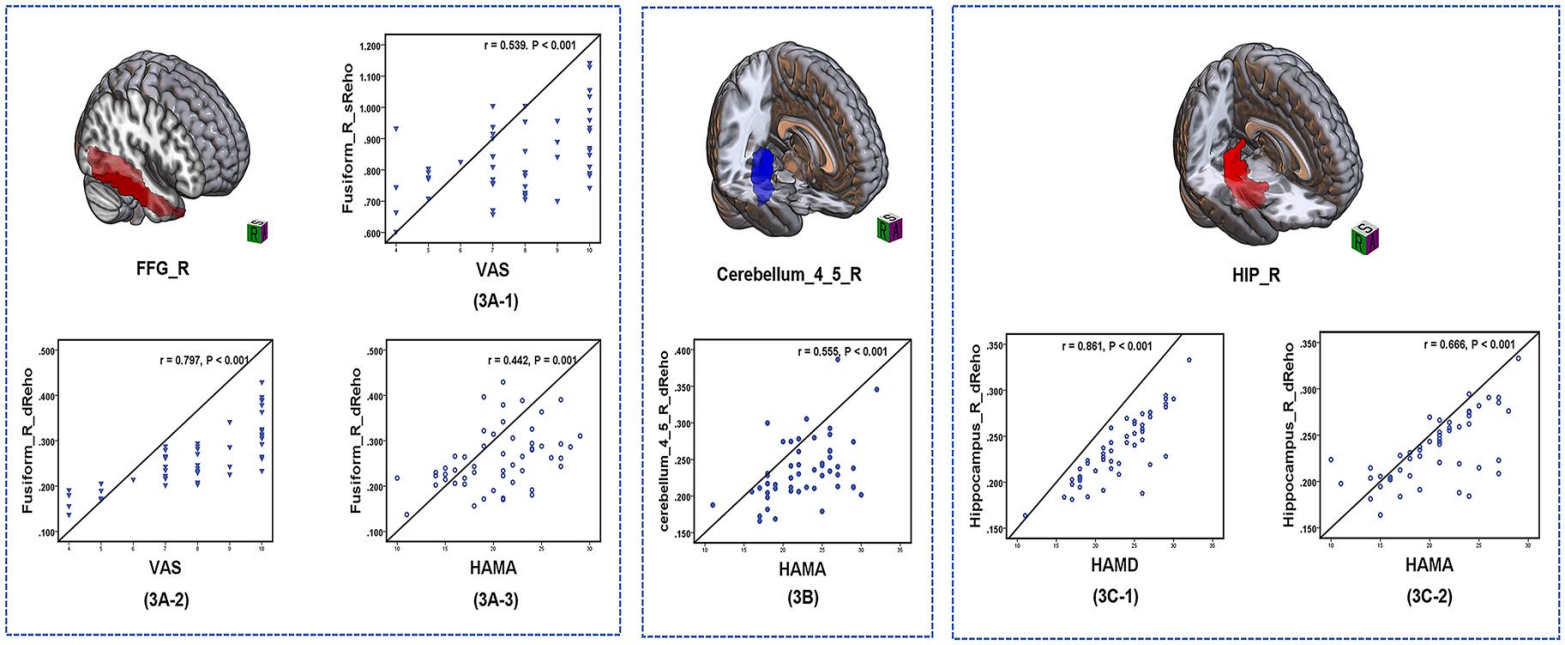
Significantly positive correlation between sReHo/dReHo values and pain and emotion scale scores in adenomyosis with dysmenorrhea (AMD) patients.

In the patient group, significant positive correlations were found between the sReHo value of the right FFG and VAS score (*r* = 0.539, *p* < 0.001) (Figure 3(A-1)), dReHo value of the right FFG and VAS score (*r* = 0.797, *p* < 0.001) (Figure 3(A-2)), dReHo value of the right FFG and HAMA score (*r* = 0.442, *p* = 0.001) (Figure 3(A-3)), dReHo value of the right cerebellum_4_5 and HAMA score (*r* = 0.555, *p* < 0.001) (Figure 3(B)), dReHo value of the right hippocampus and HAMD score (*r* = 0.861, *p* < 0.001) (Figure 3(C-1)) and dReHo value of the right hippocampus and HAMA score (*r* = 0.666, *p* < 0.001) (Figure 3(C-2)). However, serum CA125 and haemoglobin levels in patients with AM did not show a correlation with static or dReHo brain function indicators, and we initially overlooked this aspect.

## Discussion

We identified multiple brain regions exhibiting abnormal spontaneous activity and synchronization in patients with AMD based on sReHo and dReHo analysis, including the right FFG, right cerebellum, right hippocampus, left MFG, left inferior frontal gyrus (ORBinf, IFGtriang and IFGoperc) and bilateral inferior parietal lobule (SMG and ANG). Moreover, ReHo values of the hippocampus, FFG and cerebellum were significantly correlated with clinical features, strongly suggesting that regional changes in intrinsic excitability and circuit organization contribute to symptom expression and severity.

We observed significant abnormality occurred in the right hippocampus and right FFG. Previous studies [26–33] have demonstrated that modulating the expression levels of signalling proteins within the hippocampus affects pain sensitivity in mice, indicating its involvement in pain modulation. Tan et al. [34] found increased fractional anisotropy and sReHo in the right hippocampus of trigeminal neuralgia patients, while Baliki et al. [35] reported reduced grey matter density in the hippocampus of patients with various chronic pain conditions. Additionally, alterations in FFG structure have been associated with chronic pain syndromes [36–40], including greater grey matter volume as measured by VBM among patients with peripheral neuropathic pain [36], low back pain [37] and migraine with aura [39]. Ter Minassian et al. [38] identified the FFG as a key node in the default mode network activated during pain perception and found that FFG activity was negatively correlated with pain intensity rating. Our findings are consistent with previous studies, suggesting the involvement of the hippocampus and FFG in chronic pain syndromes. Furthermore, a positive correlation between FFG and pain severity implies that functional circuit abnormalities within the FFG related to pain in patients with AMD. Furthermore, positive correlations between dReHo values of the FFG and pain severity further suggest that functional circuit abnormalities within the FFG related to the pain in patients with AMD.

Abnormal spontaneous brain activity was also observed in the frontal and parietal lobes, including the left MFG, left inferior frontal gyrus (ORBinf, IFGtriang and IFGoperc) and bilateral inferior parietal lobule (SMG and ANG). These regions, interconnected and involved in cognitive functions, emotional regulation and pain encoding, have been implicated in various chronic pain disorders [41,42]. Zhang et al. [43] reported significantly elevated voxel-mirrored homotopic connectivity (VMHC) in the bilateral MFG of individuals with primary dysmenorrhea. They further observed a negative correlation between pain severity and hyperconnectivity between the left MFG and right cingulate gyrus. Dumkrieger et al. [44] detected significant correlations between years lived with headache and static FC of the left SMG, as well as between headache frequency and dynamic FC of right SMG among patients with persistent posttraumatic headache. Alhajri et al. [45] reported that dynamic ANG connectivity at alpha and beta frequencies was reduced during tonic pain, suggesting that ANG may be involved in the onset or maintenance of persistent pain states. Our research findings suggest that these abnormal local brain activities in the frontal and parietal lobes may be associated with pain in patients with AMD.

Furthermore, greater dReHo of the right cerebellum was detected in patients with AMD, consistent with previous research implicating the cerebellum in various aspects of pain processing [46–49]. Animal studies have demonstrated the cerebellum’s involvement in modulating the spinal transmission of pain signals [50,51]. A human fMRI study has linked cerebellar activity to pain perception and emotional processing [52]. Given the complex nature of pain associated with AM, characterized by central and peripheral sensitization of visceral and peritoneal nerve fibres [12], increased ReHo in the right cerebellum may reflect heightened pain sensitivity in patients with AMD.

Interestingly, we found strong and highly significant positive correlations between ReHo values and the severity of depression and anxiety symptoms as measured by the HAMA and HAMD. Previous studies [53,54] have also implicated the hippocampus, FFG and cerebellum in anxiety, depression and emotional recognition. A VBM study by Jung et al. [55] also found FFG and hippocampus volume reductions among patients with schizophrenia spectrum psychosis that were associated with impaired recognition of facial emotions and emotional intensity. A review by Stanca et al. [53] posited that the cerebellum participates in visual, auditory and self-motor perception as well as emotional processing involved in anxiety, depression and bipolar disorder. On the other hand, the cerebellum, FFG, hippocampus, inferior frontal gyrus and inferior parietal gyrus are thought to be core regions for the processing of pain-related empathy [56–58]. This suggests that visual stimulation can evoke heightened pain response and fear. Adenomyosis-associated dysmenorrhea patients are often in a state of emotional arousal, and thus susceptible to negative thoughts regarding the recurrence of menstrual pain and (or) lower abdominal pain before menstruation. Therefore, we speculate that abnormal spontaneous activation of the hippocampus, FFG, cerebellum, inferior frontal gyrus and inferior parietal gyrus may be associated with the transmission of negative emotional states (e.g. anxiety, depression and adverse empathetic psychological effects) induced by mental stress in individuals with AMD. Specifically, the increased ReHo in the hippocampus and FFG could indicate heightened pain sensitivity and enhanced emotional processing, reflecting an increased awareness of pain and emotional distress in these patients. In contrast, the decreased ReHo in regions like the prefrontal cortex may suggest reduced sensitivity to pain and emotional challenges, or maladaptive neural responses to these persistent stimuli. The distinct patterns of ReHo alterations in different brain regions may thus reflect the complex interplay between pain perception and emotional regulation in AMD patients, involving both adaptive and maladaptive neural mechanisms. This study has several limitations. First, although we excluded patients with AMD with a history of psychiatric disorders at the time of enrolment, baseline HAMA and HAMD scores were still significantly higher than in HC subjects, possibly because all patients with AMD were examined before uterine embolization surgery. Non-preoperative patients with AMD will be enrolled in future studies. Second, the duration of illness in AMD patients may influence brain function, but this factor was not systematically stratified in the current study. Future research will benefit from finer subgrouping based on disease duration to clarify its impact on ReHo alterations. Third, all AMD patients had undergone prior treatments before uterine artery embolization, which may have affected their baseline emotional state and neural activity. This preoperative condition could represent a potential confounding variable. Future investigations should aim to include treatment-naïve AMD patients and perform more granular subgroup analyses to assess the potential effects of pre-treatment disease duration, specific medication categories, treatment duration and lifestyle factors on both sReHo and dReHo patterns. Fourth, the cross-sectional study design precluded conclusions on causality. Longitudinal studies are needed to verify the reliability and reveal mechanistic implications of these findings. Fourth, AM was excluded in HC by MRI but not by pathology, so nascent or mild cases may have been included among the controls. Fifth, the absence of an a priori power analysis may have resulted in an insufficient sample size to ensure adequate statistical power. Future studies employing larger cohorts with rigorously calculated sample sizes are warranted to validate and reinforce the robustness of our findings.

## Conclusions

This study, based on sReHo and dReHo analyses, revealed abnormal local brain activity in patients with AMD which was significantly associated with pain perception and emotional disturbances (anxiety, depression). The results suggest that local brain function abnormalities may play a crucial role in chronic pain and emotional regulation impairments in AMD patients, and ReHo metrics could serve as potential neuroimaging biomarkers. ReHo reflects local neural activity but does not capture broader brain network interactions. Future research will expand on brain network analyses to better understand the role of these local abnormalities in chronic pain and emotional regulation in AMD.

## Data Availability

The data associated with this study have not been deposited in any public repository due to privacy and ethical considerations, but can be available from the corresponding author upon request.
